# Indoxyl Sulfate-Induced Oxidative Stress, Mitochondrial Dysfunction, and Impaired Biogenesis Are Partly Protected by Vitamin C and N-Acetylcysteine

**DOI:** 10.1155/2015/620826

**Published:** 2015-03-09

**Authors:** Wen-Chin Lee, Lung-Chih Li, Jin-Bor Chen, Hsueh-Wei Chang

**Affiliations:** ^1^Division of Nephrology, Department of Internal Medicine, Kaohsiung Chang Gung Memorial Hospital and Chang Gung University College of Medicine, Kaohsiung 83301, Taiwan; ^2^Institute of Medical Science and Technology, National Sun Yat-Sen University, Kaohsiung 80424, Taiwan; ^3^Center of Environmental Medicine, Kaohsiung Medical University, Kaohsiung 80708, Taiwan; ^4^Cancer Center, Kaohsiung Medical University Hospital, Kaohsiung Medical University, Kaohsiung 80708, Taiwan; ^5^Department of Biomedical Science and Environmental Biology, Kaohsiung Medical University, Kaohsiung 80708, Taiwan

## Abstract

Indoxyl sulfate (IS) contributes to oxidative stress and endothelial dysfunction in chronic kidney disease patients. However, the role of mitochondria in IS-induced oxidative stress is not very clear. In this study, we examined whether mitochondria play a pivotal role in modulating the effects of antioxidants during IS treatment. In the context of human umbilical vein endothelial cells, we found that IS had a dose-dependent antiproliferative effect. In addition, we used flow cytometry to demonstrate that the level of reactive oxygen species increased in a dose-dependent manner after treatment with IS. High doses of IS also corresponded to increased mitochondrial depolarization and decreased mitochondrial DNA copy number and mitochondrial mass. However, these effects could be reversed by the addition of antioxidants, namely, vitamin C and N-acetylcysteine. Thus, our results suggest that IS-induced oxidative stress and antiproliferative effect can be attributed to mitochondrial dysfunction and impaired biogenesis and that these processes can be protected by treatment with antioxidants.

## 1. Introduction

Indoxyl sulfate (IS) is a uremic toxin associated with vascular disease and mortality in chronic kidney disease (CKD) patients [1]. Increased reactive oxygen species (ROS) generation contributes to tissue dysfunction [2]. Moreover, IS is a known cause of oxidative stress in endothelial cells [3–5], and it has been shown to strongly decrease the levels of cellular antioxidants such as glutathione (GSH) [3] and increase the production of mitochondrial superoxide [6]. Additionally, IS has been reported to inhibit nitric oxide generation and cell proliferation in vascular endothelial cells [4].

Recently, many studies have investigated compounds that may be capable of regulating IS levels. For example, Kremezin (AST-120), an oral clinical drug with spherical adsorptive carbon, was reported to absorb IS in the gut, decreasing the IS levels in circulation in CKD patients [7]; this improved endothelial function and restored GSH levels [5]. This research provides evidence of the significant role of IS modulators in CKD patients.

Since IS is known to induce oxidative stress, it is reasonable to hypothesize that antioxidants could counteract IS-induced ROS production. Antioxidants such as vitamin E, vitamin C, and N-acetylcysteine (NAC) were reported to inhibit IS-induced ROS generation and antiproliferative effect in human umbilical vein endothelial cells (HUVECs) [3, 4]. Furthermore, several signaling pathways appear to be regulated in IS-treated cells. For example, IS inhibits nitric oxide generation and cell proliferation through ROS-mediated Nox4 overexpression in HUVECs [4]. In addition, IS upregulates intercellular adhesion molecule-1 (ICAM-1) and monocyte chemotactic protein-1 (MCP-1) expression via ROS-activated NF-*κ*B signaling [8, 9].

Mitochondria are the major ROS-generating organelles [10, 11], and mounting evidence shows that mitochondrial dysfunction may lead to many diseases [12–14]. Although IS-induced ROS generation and antiproliferative effects were reported, the role of mitochondria in this process has not been elucidated yet.

We hypothesized that IS-induced oxidative stress results in mitochondrial dysfunction and impaired mitochondrial biogenesis. To address this hypothesis, we assessed cell viability, ROS generation, mitochondrial membrane potential, mitochondrial DNA copy number, and mitochondrial mass in IS-treated HUVECs and investigated the effects of antioxidants (vitamin C and NAC) on mitochondrial function and biogenesis.

## 2. Materials and Methods

### 2.1. Cell Lines and Chemical Information

HUVECs were incubated in a humidified atmosphere with 5% CO_2_ at 37°C. Culture medium was prepared as follows: 400 mL of M199 medium (Gibco, Grand Island, NY), 100 mL of fetal bovine serum (Sigma-Aldrich, St. Louis, MO), 25000 U/vial heparin (China Chemical & Pharmaceutical, Tainan, Taiwan), 5 mL of penicillin/streptomycin (Gibco), and 7.5 mg of endothelial cell growth supplement (ECGS) (Millipore, Billerica, MA). IS, ascorbic acid, NAC, crystal violet, and dimethyl sulfoxide (DMSO) were purchased from Sigma-Aldrich, and IS, ascorbic acid, and NAC were further reconstituted to 0–250 *μ*g/mL, 200 *μ*M, and 10 mM for use in experiments, respectively.

### 2.2. Cell Viability Assay

Cell viability was measured by crystal violet assay [15]. Cells were seeded in 96-well plates (5 × 10^3^ cells/well), treated with IS at indicated concentrations (0, 50, 125, and 250 *μ*g/mL) for 48 h, and washed thrice with phosphate-buffered saline (PBS). Crystal violet reagent (0.05% in PBS) was added to these cells and the mix was incubated for 2 h at 37°C; the cells were then washed 10 times with PBS and air-dried. The cells were incubated with 100 *μ*L of DMSO for 1-2 h to completely dissolve the dye. Finally, the absorbance of these plates was read at 570 nm on a microplate reader.

### 2.3. Intracellular ROS Detection

Intracellular ROS was detected using 2′,7′-dichlorodihydrofluorescein diacetate (DCFH-DA) [16]. For this purpose, HUVECs (2 × 10^5^ cells) were incubated with different concentrations of IS (0, 50, 125, and 250 *μ*g/mL) for 48 h. Cells were then stained with 10 *μ*M DCFH-DA (Molecular Probes, Life Technologies, Carlsbad, CA) for 30 min at 37°C and detached with trypsin/EDTA. Cells were collected in PBS, washed twice by centrifugation (1500 rpm for 5 min), and resuspended in 0.5 mL of PBS. ROS production was measured by flow cytometry utilizing a fluorescence-activated cell scanner machine (BD Biosciences FACScan system). It was expressed as the mean fluorescence intensity (MFI), which was calculated by CellQuest software.

### 2.4. Measurement of Mitochondrial Membrane Potential (MMP)

Rhodamine 123 (Invitrogen, Life Technologies, Carlsbad, CA) was used to measure MMP, as described previously [17]. In brief, 2 × 10^5^ cells were plated in each well of six-well plates and they were allowed to attach for 16–18 h. After being treated with drugs for 48 h, the cells were harvested by trypsinization, washed in PBS, and resuspended in 200 ng/mL Rhodamine 123. After incubation for 30 min at 37°C, the cells were washed three times and resuspended in 500 mL of PBS. Cytofluorimetric analysis was performed using a BD Biosciences FACScan system. The fluorescence intensity of IS-treated cells was adjusted to that of the untreated control.

### 2.5. RNA Extraction and Mitochondrial DNA Copy Number

After drug treatment for 48 h, cellular RNA was extracted by PureLink RNA Mini Kit (Ambion, Life Technologies, Carlsbad, CA) and reverse-transcribed to cDNA using SuperScript First-Strand Synthesis System (Invitrogen, Life Technologies, Carlsbad, CA). Mitochondrial DNA (mtDNA) copy number was assessed using real-time polymerase chain reaction (PCR) and adjusted with nuclear DNA (*β*-actin gene) by using primers against MT-ND1 (forward: 5′-TGGGTACAATGAGGAGTAGG-3′ and reverse: 5′-GGAGTAATCCAGGTCGGT-3′) and actin (forward: 5′-TCACCCACACTGTGCCCATCTACGA-3′ and reverse: 5′-CAGCGGAACCGCTCATTGCCAATGG-3′), as described previously [18, 19]. The threshold cycle number (Ct) values of *β*-actin and ND1 genes were calculated using the following formula: relative copy number (Rc) = 2^ΔCt^, where ΔCt = Ct *β*-actin − Ct ND1.

### 2.6. Mitochondrial Mass

Mitochondrial mass was determined as described previously with slight modification [20]. In brief, the mitochondria and cytoskeleton (F-actin) were stained by MitoTracker red (Invitrogen, Life Technologies, Carlsbad, CA) and phalloidin (Molecular Probes, Life Technologies, Carlsbad, CA), respectively, and observed using a fluorescence microscope (Olympus BX-UCDB-2; Olympus Corporation, Tokyo, Japan). Image area was analyzed by the Image Pro-Plus version 7.0 (Media Cybernetics, Rockville, MD) system. Only cells with intact cytoplasmic phalloidin staining were counted. Mitochondrial mass was calculated as follows:

(1) $$\begin{array}{c} \text{mitochondrial}\;\;\text{mass}=\frac{\text{mitochondrial}\;\;\text{area}}{\text{cytoskeleton}\;\;\text{area}}\times 100. \end{array}$$

## 3. Results

### 3.1. Cell Viability of IS-Treated HUVECs

**Figure 1:**
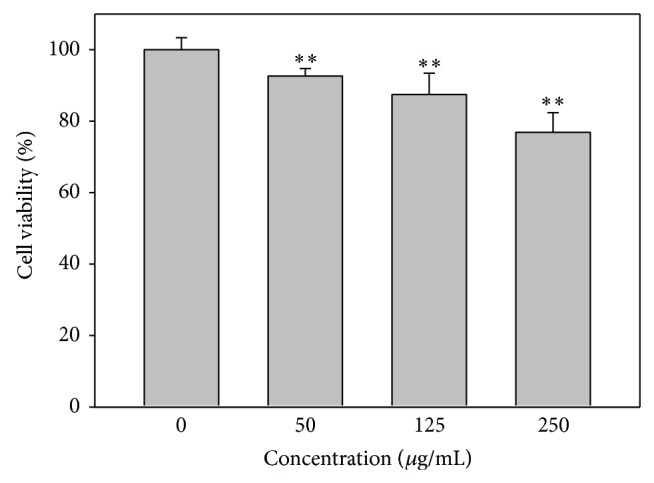
Cell viability of IS-treated HUVECs. Cells were treated with 0, 50, 125, and 250 *μ*g/mL of IS for 48 h and subjected to crystal violet assay. Data, mean ± SD (*n* = 12). ^**^ *P* < 0.0001.

The viability of HUVECs treated with the indicated concentrations of IS for 48 h is shown in Figure 1. Cell viability was found to be inversely correlated with IS dose ranging from 0 to 250 *μ*g/mL. In addition, the antiproliferative effect of IS on HUVECs significantly increased in a dose-dependent manner (Figure 1).

### 3.2. ROS Generation of IS-Treated HUVECs

**Figure 2:**
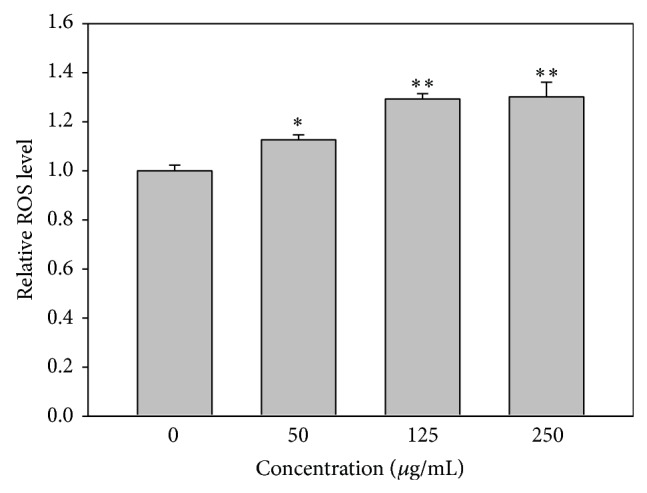
ROS generation of IS-treated HUVECs. Quantification analysis of relative ROS mean intensity of DCFH-DA (% of control). Cells were treated with 0, 50, 125, and 250 *μ*g/mL of IS for 24 h. Data, mean ± SD (*n* = 3). ^*^ *P* < 0.01; ^**^ *P* < 0.001 compared to the control.

The mean fluorescence intensity of DCFH-DA was used to measure the relative ROS content (% of control) in IS-treated HUVECs. The results indicated that increasing doses of IS corresponded to higher levels of ROS (Figure 2).

### 3.3. MMP of IS-Treated HUVECs

**Figure 3:**
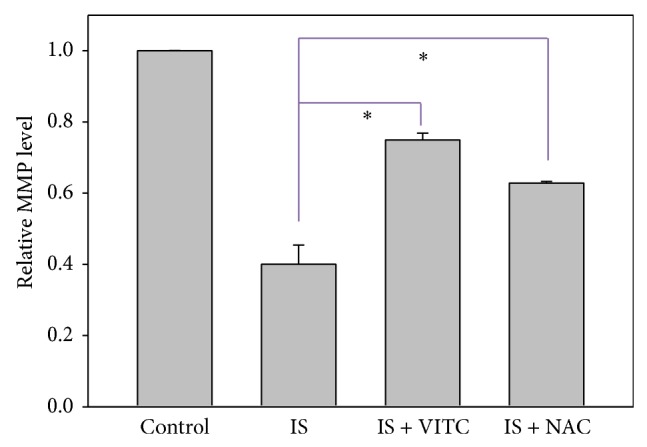
Role of antioxidants in IS-induced mitochondrial depolarization in HUVECs. Quantification analysis of relative MMP mean intensity (% of control) of Rhodamine 123. Cells were treated with IS 125 *μ*g/mL alone as the positive control, cotreated with IS 125 *μ*g/mL and vitamin C 200 *μ*M or NAC 10 mM for 48 h. Cells without IS treatment were regarded as the negative control. Data, mean ± SD (*n* = 2). ^*^ *P* < 0.05 compared to the control.

The mean fluorescence intensity of Rhodamine 123 was used to measure the relative MMP levels (% of control) in IS-treated HUVECs.  Figure 3 shows that MMP was reduced in IS-treated HUVECs. However, the addition of antioxidants such as vitamin C or NAC was able to counteract the effect of IS with regard to MMP (Figure 3).

### 3.4. Mitochondrial Function in IS-Treated HUVECs

Mitochondrial DNA (mtDNA) expression was measured to quantify mitochondrial function in HUVECs treated with IS. In accordance with our previous findings, we saw that mtDNA copy number was dramatically reduced in IS-treated HUVECs, compared to untreated controls. Moreover, these effects could be reversed by the addition of either vitamin C or NAC.

### 3.5. Mitochondrial Biogenesis in IS-Treated HUVECs

We assessed the effect of IS treatment on mitochondrial biogenesis by measuring mitochondrial mass in IS-treated HUVECs. To this end, we stained the mitochondria, cytoskeletal networks, and nuclei by using MitoTracker red, phalloidin, and DAPI, respectively. Using imaging software, we were able to quantify the mitochondrial mass relative to that of the entire cell (Figure 5(b)); increase in the number of loci of red fluorescent staining indicates increased mitochondrial mass in the cytoplasm. Our results show that mitochondrial mass was dramatically reduced in IS-treated HUVECs compared to controls and that we could counteract these effects with the addition of vitamin C or NAC.

## 4. Discussion

IS is a known risk factor for cardiovascular disease in CKD patients [1, 21]. Findings from the present and previous studies [3–5] supported the idea that IS can induce antiproliferative effects and ROS generation in a dose-dependent manner. ROS generation was commonly reported, along with mitochondrial depolarization and apoptosis, for example, cancer cells treatment with natural products [22–24]. Similarly, we found that IS induced mitochondrial depolarization and cell death, although the cause of death was not verified to be apoptosis. Moreover, the sensitivity of mitochondria-based methods for cell viability detection like MTT or MTS [23, 24] is better than the crystal violet assay used in the current study. For small dosage effect of IS, the MTT/MTS assays can be considered.

Vitamin C [25] and NAC [26] are both well-known scavengers of free radicals. Interestingly, oxidative stress associated with other ROS-generating drugs such as imidacloprid [27] and melamine [28] was shown to be thwarted by treatment with vitamin C. In addition, liver carcinogenesis induced by diethylnitrosamine was mediated by ROS generation and was reversed by NAC in animal models [29]. Furthermore, pretreatment with NAC partly rescued IS-induced antiproliferative effects and nitric oxide generation in HUVECs at 48 h IS treatment [5]. In our study, we focused on the effect of IS on mitochondrial dysfunction and found that both vitamin C and NAC significantly protected HUVECs from IS-induced mitochondrial dysfunction.

The ROS production of HUVECs stimulated by IS was dose dependent. However, IS could activate NADPH oxidase (NOX) to increase ROS production [3]. It could also relate to mitochondria dysfunction. Accordingly, the origin of ROS production (NOX or mitochondria) after adding IS to HUVECs remains unclear. Using NOX inhibitors [3] or p22phox siRNA [30] for NOX and monitoring cytochrome C oxidase activity may further address this issue.

**Figure 4:**
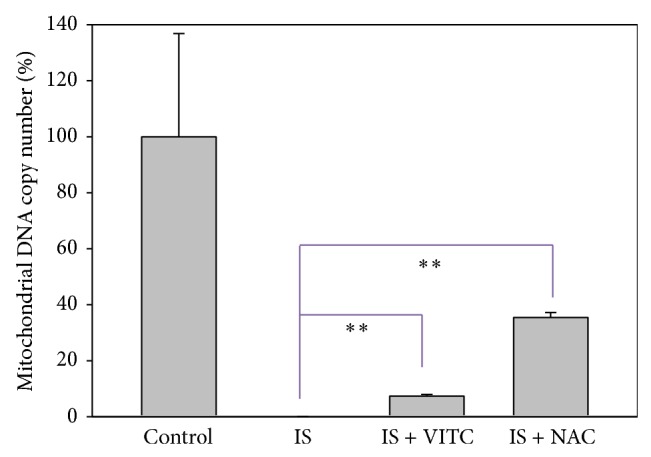
Role of antioxidants in IS-induced mtDNA copy number changes in HUVECs. Quantification analysis of relative mtDNA copy number (% of control) was shown. Cells were treated with IS 125 *μ*g/mL alone as the positive control, cotreated with IS 125 *μ*g/mL and vitamin C 200 *μ*M or NAC 10 mM for 48 h. Cells without IS treatment were regarded as the negative control. Data, mean ± SD (*n* = 2). ^**^ *P* < 0.005 compared to the IS only. VITC: vitamin C.

**Figure 5:**
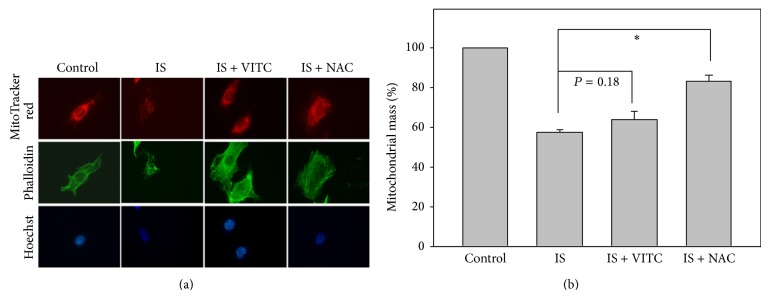
Role of antioxidants in IS-induced mitochondrial mass changes in HUVECs. Cells were treated with IS 125 *μ*g/mL alone as the positive control, cotreated with IS 125 *μ*g/mL and vitamin C 200 *μ*M or NAC 10 mM for 48 h. Cells without IS treatment were regarded as the negative control. The fluorescent dyes and colors were MitoTracker (red), phalloidin (green), and Hoechst 33258 (blue). (a) Representative confocal microscopy. (b) Quantification analysis of relative mitochondrial mass (% of control). Data, mean ± SD (*n* = 2). ^*^ *P* < 0.01 compared to the IS only.

Many researchers have quantified mitochondrial biogenesis in terms of mitochondrial DNA copy number and mitochondrial mass. For example, knockdown of manganese superoxide dismutase (MnSOD) was reported to increase mtDNA copy number and mitochondrial mass in normal rat kidney cells [31], and overexpression of mitochondrial transcription factor A (TFAM) increased mtDNA copy number and preserved transformation-induced oxidative stress in lymphoblastoid cells [32]. Here, we have shown that IS reduced mtDNA copy number and mitochondrial mass in HUVECs and that these results could be reversed with the addition of vitamin C and NAC. However, some valuable assays including mtDNA deletion assay [33, 34], may offer complementary information on how much effect that IS had on mtDNA reduction. Moreover, the possible presence of the large mtDNA deletions may partly explain that IS-induced marked reduction of PCR-based mtDNA copy number to low level (Figure 4) while the IS-induced mitochondrial mass seems to maintain stable level (Figure 5).

Other antioxidants may have the potential to improve mitochondrial function and biogenesis. For example, red wine polyphenols such as resveratrol enhance mitochondrial biogenesis in coronary arterial endothelial cells via sirtuin 1 activation [20]. Interestingly, other members of the sirtuin family are linked to ROS generation [35]; sirtuin 2 overexpression induced ROS generation in non-small-cell lung cancer [36], and sirtuin 2 was reported to induce alveolar mitochondrial biogenesis in the animal model of* Staphylococcus aureus* pneumonia [37]. These findings indicate the potential roles of ROS-signaling proteins in the regulation of mitochondrial biogenesis.

Recently, many mitochondrial biogenesis-related transcription factors have been identified. These include peroxisome proliferator-activated receptor gamma coactivator 1-alpha (PGC-1*α*; PPARGC1A) [38], mitochondrial transcription factors A (TFAM), B1 (TFB1M), and B2 (TFB2M) [39], nuclear respiratory factor 1 (NRF1) [40], nuclear factor, erythroid 2-like 2 (NRF2; NFE2L2) [37], and estrogen-related receptor-*α* (ESRRA) [41]. Further research is necessary to examine alterations in the expression of these transcription factors in IS-treated cells. This will offer better understanding of the molecular networks that are involved in helping human endothelial cells cope with IS-mediated injuries.

## References

[1] Barreto F. C., Barreto D. V., Liabeuf S. (2009). Serum indoxyl sulfate is associated with vascular disease and mortality in chronic kidney disease patients. *Clinical Journal of the American Society of Nephrology*.

[2] Ruan X. Z., Varghese Z., Moorhead J. F. (2009). An update on the lipid nephrotoxicity hypothesis. *Nature Reviews Nephrology*.

[3] Dou L., Jourde-Chiche N., Faure V. (2007). The uremic solute indoxyl sulfate induces oxidative stress in endothelial cells. *Journal of Thrombosis and Haemostasis*.

[4] Tumur Z., Niwa T. (2009). Indoxyl sulfate inhibits nitric oxide production and cell viability by inducing oxidative stress in vascular endothelial cells. *American Journal of Nephrology*.

[5] Yu M., Kim Y. J., Kang D. H. (2011). Indoxyl sulfate-induced endothelial dysfunction in patients with chronic kidney disease via an induction of oxidative stress. *Clinical Journal of the American Society of Nephrology*.

[6] Owada S., Maeba T., Sugano Y. (2010). Spherical carbon adsorbent (AST-120) protects deterioration of renal function in chronic kidney disease rats through inhibition of reactive oxygen species production from mitochondria and reduction of serum lipid peroxidation. *Nephron Experimental Nephrology*.

[7] Shimoishi K., Anraku M., Kitamura K. (2007). An oral adsorbent, AST-120 protects against the progression of oxidative stress by reducing the accumulation of indoxyl sulfate in the systemic circulation in renal failure. *Pharmaceutical Research*.

[8] Tumur Z., Shimizu H., Enomoto A., Miyazaki H., Niwa T. (2010). Indoxyl sulfate upregulates expression of ICAM-1 and MCP-1 by oxidative stress-induced NF-*κ*B activation. *American Journal of Nephrology*.

[9] Shimizu H., Yisireyili M., Higashiyama Y., Nishijima F., Niwa T. (2013). Indoxyl sulfate upregulates renal expression of ICAM-1 via production of ROS and activation of NF-*κ*B and p53 in proximal tubular cells. *Life Sciences*.

[10] Chen Q., Vazquez E. J., Moghaddas S., Hoppel C. L., Lesnefsky E. J. (2003). Production of reactive oxygen species by mitochondria: central role of complex III. *Journal of Biological Chemistry*.

[11] Murphy M. P. (2009). How mitochondria produce reactive oxygen species. *Biochemical Journal*.

[12] Che R., Yuan Y., Huang S., Zhang A. (2014). Mitochondrial dysfunction in the pathophysiology of renal diseases. *American Journal of Physiology—Renal Physiology*.

[13] Tang X., Luo Y. X., Chen H. Z., Liu D. P. (2014). Mitochondria, endothelial cell function, and vascular diseases. *Frontiers in Physiology*.

[14] Hroudová J., Singh N., Fišar Z. (2014). Mitochondrial dysfunctions in neurodegenerative diseases: relevance to alzheimer's disease. *BioMed Research International*.

[15] Au W., Pathak S., Collie C. J., Hsu T. C. (1978). Cytogenetic toxicity of gentian violet and crystal violet on mammalian cells in vitro. *Mutation Research*.

[16] Yeh C.-C., Yang J.-I., Lee J.-C. (2012). Anti-proliferative effect of methanolic extract of *Gracilaria tenuistipitata* on oral cancer cells involves apoptosis, DNA damage, and oxidative stress. *BMC Complementary and Alternative Medicine*.

[17] Lewis-Wambi J. S., Kim H. R., Wambi C. (2008). Buthionine sulfoximine sensitizes antihormone-resistant human breast cancer cells to estrogen-induced apoptosis. *Breast Cancer Research*.

[18] Weng S.-W., Lin T.-K., Liou C.-W. (2009). Peripheral blood mitochondrial DNA content and dysregulation of glucose metabolism. *Diabetes Research and Clinical Practice*.

[19] Liou C.-W., Lin T.-K., Chen J.-B. (2010). Association between a common mitochondrial DNA D-loop polycytosine variant and alteration of mitochondrial copy number in human peripheral blood cells. *Journal of Medical Genetics*.

[20] Csiszar A., Labinskyy N., Pinto J. T. (2009). Resveratrol induces mitochondrial biogenesis in endothelial cells. *American Journal of Physiology—Heart and Circulatory Physiology*.

[21] Menon V., Gul A., Sarnak M. J. (2005). Cardiovascular risk factors in chronic kidney disease. *Kidney International*.

[22] Yen C.-Y., Chiu C.-C., Haung R.-W. (2012). Antiproliferative effects of goniothalamin on Ca9-22 oral cancer cells through apoptosis, DNA damage and ROS induction. *Mutation Research: Genetic Toxicology and Environmental Mutagenesis*.

[23] Yeh C. C., Tseng C. N., Yang J. I. (2012). Antiproliferation and induction of apoptosis in Ca9-22 oral cancer cells by ethanolic extract of Gracilaria tenuistipitata. *Molecules*.

[24] Chiu C.-C., Haung J.-W., Chang F.-R. (2013). Golden berry-derived 4*β*-hydroxywithanolide E for selectively killing oral cancer cells by generating ROS, DNA damage, and apoptotic pathways. *PLoS ONE*.

[25] Bagchi D., Garg A., Krohn R. L., Bagchi M., Tran M. X., Stohs S. J. (1997). Oxygen free radical scavenging abilities of vitamins C and E, and a grape seed proanthocyanidin extract in vitro. *Research Communications in Molecular Pathology and Pharmacology*.

[26] Zafarullah M., Li W. Q., Sylvester J., Ahmad M. (2003). Molecular mechanisms of *N*-acetylcysteine actions. *Cellular and Molecular Life Sciences*.

[27] EL-Gendy K. S., Aly N. M., Mahmoud F. H., Kenawy A., El-Sebae A. K. H. (2010). The role of vitamin C as antioxidant in protection of oxidative stress induced by imidacloprid. *Food and Chemical Toxicology*.

[28] An L., Li Z., Zhang T. (2014). Reversible effects of vitamins C and E combination on oxidative stress-induced apoptosis in melamine-treated PC12 cells. *Free Radical Research*.

[29] Lin H., Liu X.-B., Yu J.-J., Hua F., Hu Z.-W. (2013). Antioxidant N-acetylcysteine attenuates hepatocarcinogenesis by inhibiting ROS/ER stress in TLR2 deficient mouse. *PLoS ONE*.

[30] Sardina J. L., López-Ruano G., Sánchez-Abarca L. I. (2010). P22phox-dependent NADPH oxidase activity is required for megakaryocytic differentiation. *Cell Death and Differentiation*.

[31] Marine A., Krager K. J., Aykin-Burns N., MacMillan-Crow L. A. (2014). Peroxynitrite induced mitochondrial biogenesis following MnSOD knockdown in normal rat kidney (NRK) cells. *Redox Biology*.

[32] Chakrabarty S., D'Souza R. R., Kabekkodu S. P., Gopinath P. M., Rossignol R., Satyamoorthy K. (2014). Upregulation of TFAM and mitochondria copy number in human lymphoblastoid cells. *Mitochondrion*.

[33] Kraytsberg Y., Kudryavtseva E., McKee A. C., Geula C., Kowall N. W., Khrapko K. (2006). Mitochondrial DNA deletions are abundant and cause functional impairment in aged human substantia nigra neurons. *Nature Genetics*.

[34] Majora M., Wittkampf T., Schuermann B. (2009). Functional consequences of mitochondrial DNA deletions in human skin fibroblasts: increased contractile strength in collagen lattices is due to oxidative stress-induced lysyl oxidase activity. *American Journal of Pathology*.

[35] Merksamer P. I., Liu Y., He W., Hirschey M. D., Chen D., Verdin E. (2013). The sirtuins, oxidative stress and aging: an emerging link. *Aging*.

[36] Li Z., Xie Q. R., Chen Z., Lu S., Xia W. (2013). Regulation of SIRT2 levels for human non-small cell lung cancer therapy. *Lung Cancer*.

[37] Athale J., Ulrich A., Chou MacGarvey N. (2012). Nrf2 promotes alveolar mitochondrial biogenesis and resolution of lung injury in Staphylococcus aureus pneumonia in mice. *Free Radical Biology and Medicine*.

[38] Ventura-Clapier R., Garnier A., Veksler V. (2008). Transcriptional control of mitochondrial biogenesis: the central role of PGC-1*α*. *Cardiovascular Research*.

[39] Falkenberg M., Gaspari M., Rantanen A., Trifunovic A., Larsson N.-G., Gustafsson C. M. (2002). Mitochondrial transcription factors B1 and B2 activate transcription of human mtDNA. *Nature Genetics*.

[40] Tiranti V., Rossi E., Rocchi M., DiDonato S., Zuffardi O., Zeviani M. (1995). The gene (NFE2L1) for human NRF-1, an activator involved in nuclear-mitochondrial interactions, maps to 7q32. *Genomics*.

[41] Schreiber S. N., Emter R., Hock M. B. (2004). The estrogen-related receptor *α* (ERR*α*) functions in PPAR*γ* coactivator 1*α* (PGC-1*α*)-induced mitochondrial biogenesis. *Proceedings of the National Academy of Sciences of the United States of America*.

